# Dr. Henry Clay Baum

**Published:** 1916-09

**Authors:** 


**Dr. Henry Clay Baum,** Michigan 1883, a prominent practitioner of Syracuse, died Aug. 6.

